# Chirality-Induced Spin Selectivity in Supramolecular Chirally Functionalized Graphene

**DOI:** 10.1021/acsnano.3c06903

**Published:** 2023-09-05

**Authors:** Seyedamin Firouzeh, Sara Illescas-Lopez, Md Anik Hossain, Juan Manuel Cuerva, Luis Álvarez de Cienfuegos, Sandipan Pramanik

**Affiliations:** † Department of Electrical and Computer Engineering, University of Alberta, Edmonton, Alberta T6G 1H9, Canada; ‡ Universidad de Granada, Departamento de Química Orgánica, Unidad de Excelencia Química Aplicada a Biomedicina y Medioambiente, C. U. Fuentenueva, Avda. Severo Ochoa s/n, E-18071 Granada, Spain; § Instituto de Investigación Biosanitaria ibs., Avda. De Madrid, 15, E-18016 Granada, Spain

**Keywords:** chirality-induced spin selectivity, graphene, supramolecular chirality, carbon nanosheets, short peptides, spin transport

## Abstract

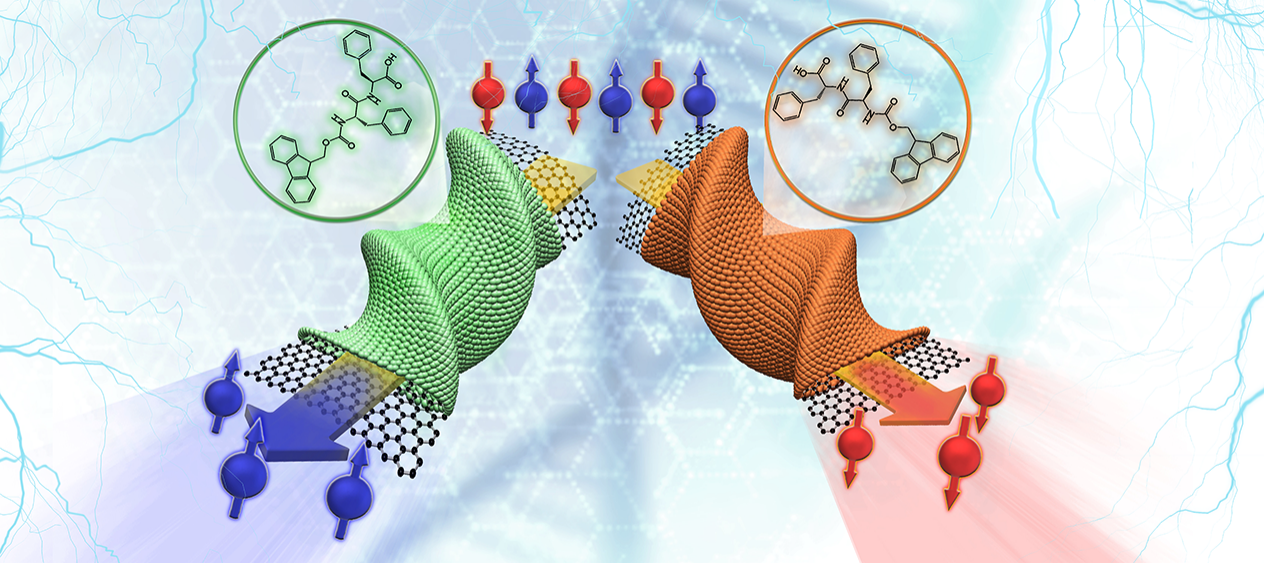

Chiral graphene hybrid materials have attracted significant attention in recent years due to their various applications in the areas of chiral catalysis, chiral separation and recognition, enantioselective sensing, etc. On the other hand, chiral materials are also known to exhibit chirality-dependent spin transmission, commonly dubbed “chirality induced spin selectivity” or CISS. However, CISS properties of chiral graphene materials are largely unexplored. As such, it is not clear whether graphene is even a promising material for the CISS effect given its weak spin–orbit interaction. Here, we report the CISS effect in chiral graphene sheets, in which a graphene derivative (reduced graphene oxide or rGO) is noncovalently functionalized with chiral Fmoc-FF (Fmoc-diphenylalanine) supramolecular fibers. The graphene flakes acquire a “conformational chirality” postfunctionalization, which, combined with other factors, is presumably responsible for the CISS signal. The CISS signal correlates with the supramolecular chirality of the medium, which depends on the thickness of graphene used. Quite interestingly, the noncovalent supramolecular chiral functionalization of conductive materials offers a simple and straightforward methodology to induce chirality and CISS properties in a multitude of easily accessible advanced conductive materials.

## Introduction

Chirality-induced spin selectivity (CISS) refers to a set of phenomena in which chirality of a material imparts significant spin selectivity to various electronic processes.^1,2^ In the context of spintronic devices, it means generation of chirality-dependent spin polarization, when a population of spin unpolarized electrons is transmitted through a chiral medium, or detection of spins using chiral materials. Thus, chiral media can act as spin polarizers or analyzers, which are critical components of spintronic devices. The physical origin of CISS is still under debate, although it is generally assumed that the spin–orbit interaction of the medium plays a critical role.^1^

Many organic molecules such as DNA, amino acids, peptides, helicenes, etc. are inherently chiral, and hence they are commonly employed for CISS studies.^2−6^ However, these molecules are electrical insulators, which pose challenges for their integration into electronic devices and circuits. In addition, the formation of reliable electrical contacts with molecules gives rise to additional difficulties. On the other hand, various inorganic materials that are considered promising platforms for future electronics and spintronics are generally nonchiral, and hence these materials do not exhibit the CISS effect in their natural forms. Some alternative approaches have been investigated to bridge this gap. For example, it has been reported that CISS can be induced in carbon nanotubes by attaching chiral molecules on the tube walls.^7−14^ Several studies on chiral inorganic crystals have also been reported.^15−18^ However, rational integration of chiral organic materials with emerging advanced materials with promising electronic and spintronic properties is expected to significantly expand the application of CISS in the area of solid-state spintronics. Therefore, the development of strategies that allow interesting materials to be endowed with chirality in a simple and effective way is a highly desired goal in this field.

Graphene, in its pristine form, is a particularly interesting 2D material because of its unique band structure, high carrier mobility, and ability to show various quantum transport phenomena even at room temperature.^19,20^ From the viewpoint of spintronics, room-temperature spin transport and micron-scale spin relaxation length in pristine graphene layers have been reported.^21,22^ Such features originate due to the weak intrinsic spin–orbit interaction of graphene and the weak hyperfine interaction of electron spin with carbon nuclei, which suppress the spin relaxation mechanisms. On the other hand, it has been reported that various chiral organic molecules can impart chirality to various graphene derivatives such as graphene oxide (GO) and reduced (or deoxygenated) graphene oxide (rGO) via covalent as well as noncovalent interactions including hydrophobic effect, π–π stacking, electrostatic effect, etc.^23−30^ In most cases, the chirality of the resultant hybrid materials originates from the chirality of the attached moiety. These have been classified as “configurational chirality” (originating from small chiral molecules), “conformational chirality” (due to helical polymers), or “hierarchical chirality” (due to chiral liquid crystal structures).^26^ Reference (31) reported the synthesis of chiral graphene quantum dots via functionalization with chiral cysteine molecules. The molecules attach with the edges of the graphene flakes and induce “structural chirality” in the form of helical buckling of the flakes. Several application areas of chiral graphene have been explored by the above studies, which include chiral catalysis,^27^ chiral separation and recognition,^25,28,29^ enantioselective sensing,^24,30^ etc. Use of GO and rGO in these studies is motivated by the fact that they are amenable to chiral functionalization via chemical means, and chiral graphene can be generated in large quantities via solution processing, which is difficult to achieve using pristine graphene. While electrical conductivity of rGO is significantly lower than that of pristine graphene, it is still much more conductive than pure organics and commonly finds applications in field-effect transistors,^32^ chemical sensors,^33^ and conductive electrodes in various devices.^34^

At the present stage, very little, if any, is known about the CISS properties of these chiral graphene materials. *A priori*, the CISS effect is expected to be weak due to the weak spin–orbit interaction of graphene, even though rGO systems are structurally distinct from pristine graphene layers, due to the presence of defects in the form of carbon vacancies and adatoms such as hydrogen and oxygen. In any case, if the CISS effect exists in chiral rGO systems, it could potentially be used as a detection mechanism for the above chiral separation, recognition, and sensing applications.

In this work, we report the CISS effect in a thin graphene film, which consists of rGO flakes noncovalently functionalized with chiral supramolecular fibers made by the self-assembly of Fmoc-FF (L/D). Graphene is covered by the hydrophobic peptide fibers in which the aromatic rings of Fmoc-FF molecules bind efficiently with the planar surface of graphene via the π-π interaction. Control experiments are performed with Fmoc-GG (Fmoc-diglycine) molecules, which are achiral. As described below, the chiral molecules self-assemble in helical supramolecular structures along with the attached graphene and induce the CISS effect in two-terminal magnetotransport measurements. The role of graphene flake thickness has been investigated by using single-layer graphene (SLG) and multilayer graphene (MLG) flakes (both in rGO form).

## Results and Discussions

**Figure 1:**
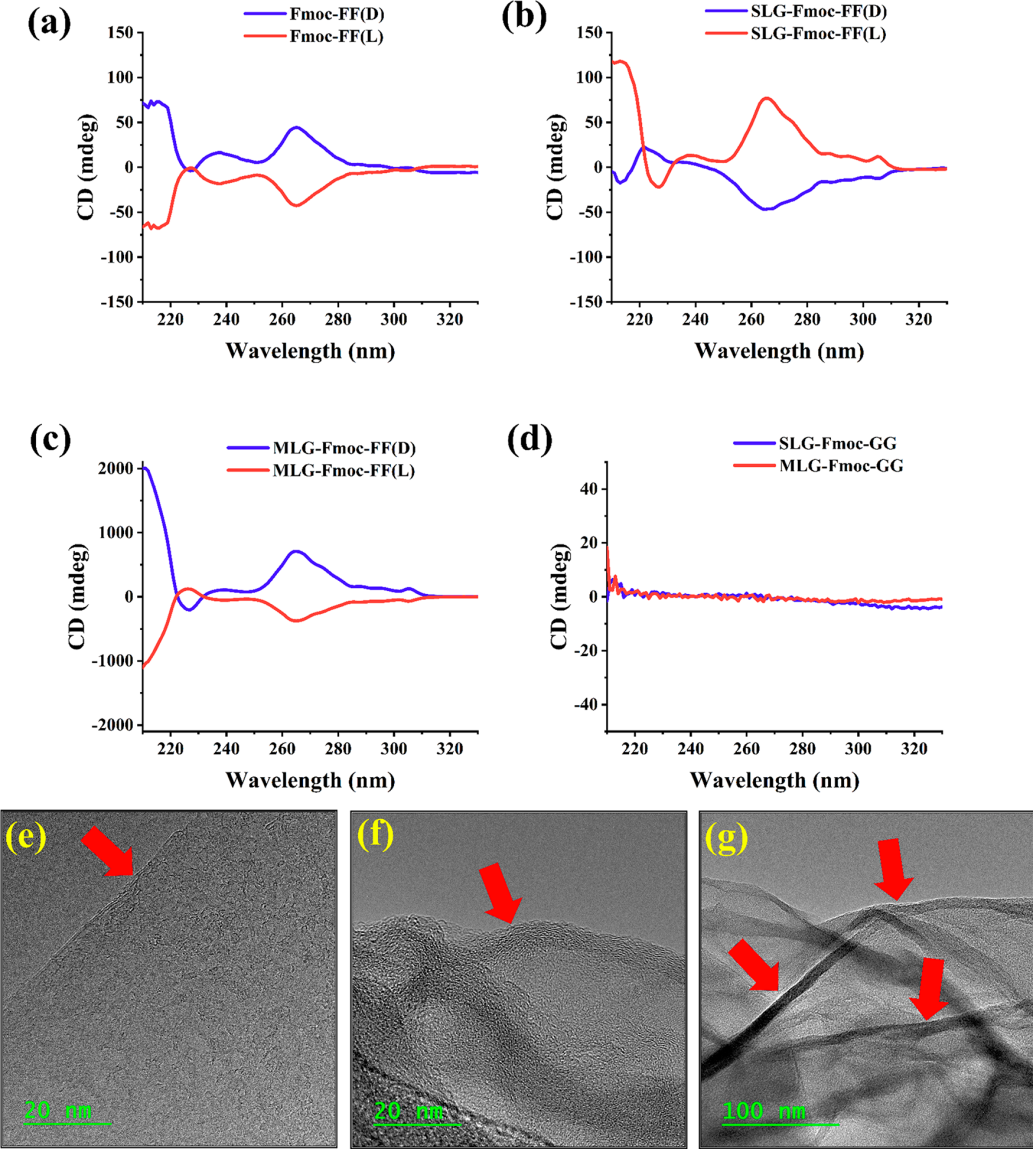
(a–d) CD characterizations of the Fmoc-FF and functionalized SLG and MLG solutions. (e) TEM image of a pristine (unfunctionalized) graphene flake. Arrow shows the straight edge of the flake. (f,g) TEM images of functionalized flakes. The arrows show the curved flakes.

Figure 1a shows the circular dichroism (CD) spectrum of the chiral (Fmoc-FF L/D) solutions. Figure 1b,c show the CD spectra of the SLG and MLG solutions, respectively, functionalized with Fmoc FF L/D molecules. Figure 1d shows the CD spectrum of the SLG and MLG solutions functionalized with achiral (Fmoc-GG) control molecules. Corresponding HT spectra are shown in Figure S2 (Supporting Information).

CD spectra of Fmoc-FF supramolecular fibers showed mirror images for both enantiomers, indicating that the chirality of the supramolecular aggregates is dictated by the intrinsic chirality of the peptide. These aggregates show two characteristic Cotton bands, one around 220 nm which corresponds to n−π* transition of the amino acids and another band around 260–270 nm which corresponds to the π–π* transition of the fluorenyl groups and was previously justified as superhelical arrangements of these peptides.^35−37^ This band determines the supramolecular arrangement of these peptides and therefore is the one related to the CISS effect.^12,13^

The self-assembly of Fmoc-FF in water is mainly mediated by hydrophobic interactions between peptides. This process can be triggered by different stimuli, and once the self-assembly is promoted, the formation of fibers is highly favored.^38^ As such, when solid particles are present in the media, they are engulfed by the peptide fibers in formation, including them in the supramolecular peptide structure and giving rise to hybrid materials.^11−14,39−41^ This process favors a strong interaction between the peptide fibers and the solid material that ends up with its surface completely functionalized with the peptides. Moreover, if the solid material is flexible, it can suffer morphological alterations in its structure, the peptide fibers acting as a template.^42,43^ In this process, the supramolecular arrangement of the peptides can also be altered by the interaction of the peptides with the solid particles, resulting in some cases in an inversion of the supramolecular chirality, as observed by changes in the sign of the Cotton bands in CD spectra. We have previously observed this effect when the self-assembly of Fmoc-FF in the presence of SWCNT (single-walled carbon nanotubes) is triggered by Na_2_CO_3_ or GdL^12^ or in the presence of CNTs of different diameters.^14^ In this case, we observed the same effect upon passing from the peptide solutions (Figure 1a) to the peptides containing SLG (Figure 1b). These samples show an inversion of the supramolecular chirality of the peptides; the π–π* transition of the fluorenyl groups has positive values for SLG-Fmoc-FF (L) and negative ones for SLG-Fmoc-FF (D). However, this inversion of supramolecular chirality is not observed when MLG is used (Figure 1c). In this case, the sign of the CD for the hybrid MLG-Fmoc-FF aggregates are preserved with respect to the Fmoc-FF solutions. Finally, graphene flakes functionalized with achiral Fmoc-GG show no CD as expected (Figure 1d).

TEM images of the SLG flakes are shown in Figure 1e–g. Figure 1e shows a reference pristine graphene flake without any functionalization. It is important to note that the pristine flake appears flat, with sharp, straight edges. In contrast, the functionalized (using Fmoc-FF D) flake shown in Figure 1f appears to be curved, resembling a partially rolled structure. Figure 1g shows the partially rolled functionalized graphene flakes on a larger scale. As commented upon above, this distortion is exerted by the peptide self-assembly process.

**Figure 2:**
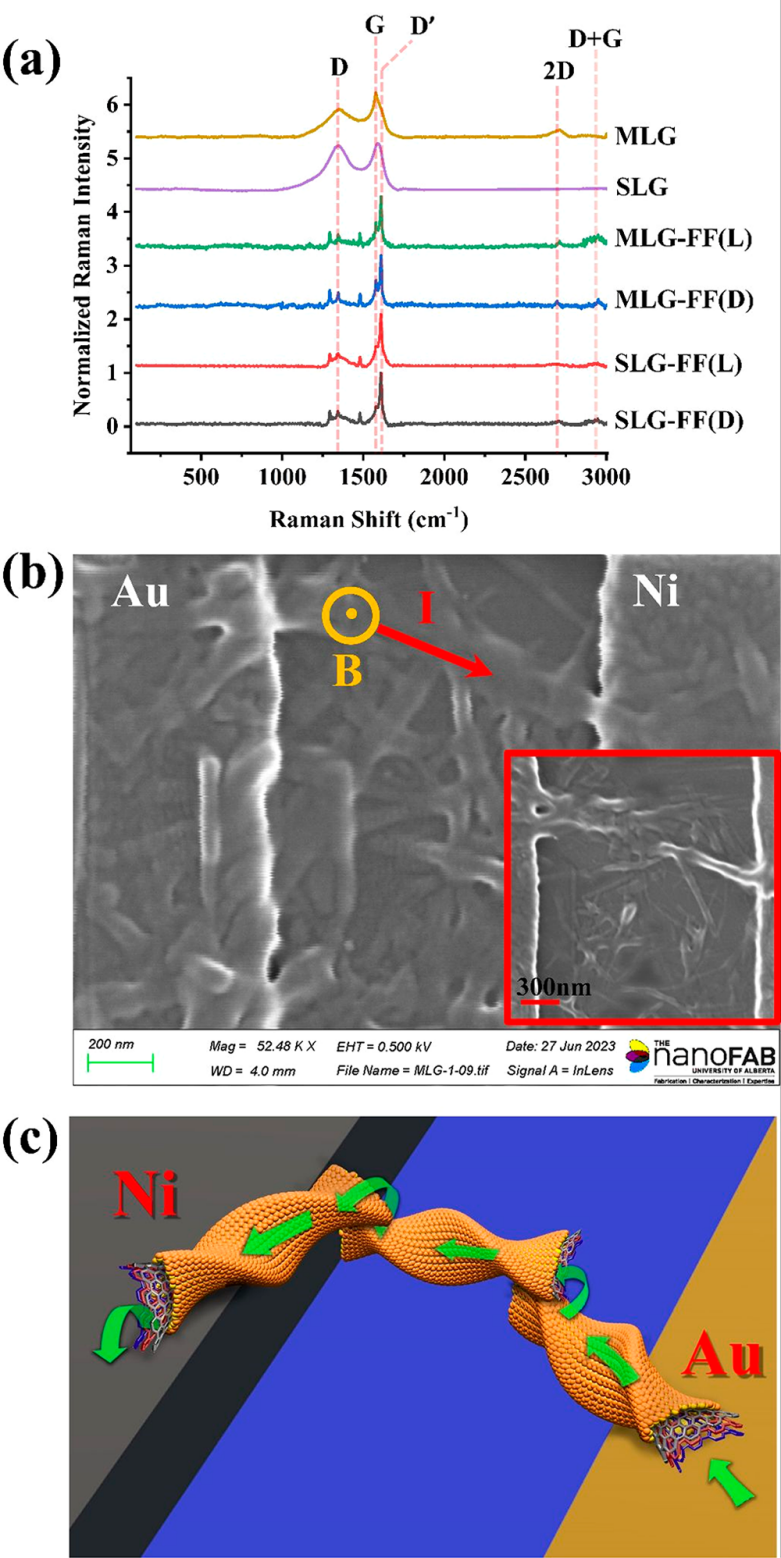
(a) Raman characterization (532 nm) of SLG and MLG, showing the relevant peaks before and after functionalization. (b) FESEM image of a two-terminal device in which a chiral-functionalized graphene layer is contacted by Au and Ni electrodes. Applied magnetic field is out-of-plane. (c) Schematic of a typical current path through the chiral graphene flakes, which have both in-plane (primary) and out-of-plane (secondary) components.

Raman spectra of raw and functionalized graphene flakes, measured in ambient air with an excitation wavelength of 532 nm, are shown in Figure 2a. At lower wavenumbers (<2000 cm^–1^), the well-known *D* (∼1350 cm^–1^), *G* (∼1580 cm^–1^), and *D*′ (∼1610 cm^–1^) peaks are present.^44^ While the strongest peak in unfunctionalized (raw) SLG and MLG is the *G* peak, the *D*′ peak appears as the dominant peak in the functionalized graphene samples. The appearance of the strong *D*′ peak after functionalization indicates the presence of defects and structural disorder. It should be noted that the *D*′ peak is relatively weak and can be challenging to distinguish from background noise in the raw samples; however, the presence of a strong *D*′ peak in conjunction with the *D* and *G* peaks after functionalization indicates lattice distortion. As commented upon above, this can arise from the self-assembly of the peptide molecules, which inflicts strain on the attached SLG and MLG layers, as indicated by the twisted and rolled layers of graphene in Figure 1f,g. In the functionalized samples, we observe additional peaks at ∼1479 cm^–1^, which is associated with the functionalization-induced structural changes and the vibrational modes of the functional groups,^45^ and also at ∼1294 cm^–1^, which is related to the oxygen-containing functional groups and carboxyl (−COOH) groups.^46^ In addition, we observe weak Raman active bands such as 2D (∼2710 cm^–1^) and D+G (∼2950 cm^–1^) at higher wavenumbers. The presence of defects and disorders broadens and weakens the 2D peak significantly, which is a characteristic of rGO.^47^

A two-terminal planar CISS device is fabricated by placing a slice of the gel described above between Au–Ni contacts. The device is vacuum annealed to improve the electrical contacts as well as to improve the connectivity between the individual chiral flakes.^48^ A FESEM image of the final device is shown in Figure 2b. The “rolled-up” graphene structures discussed previously are also visible in this image. The contact dimensions are chosen such that the intrinsic resistance of the chiral graphene layer dominates. As evident from the above fabrication process, the graphene film is not homogeneous but is composed of multiple graphene flakes, each of which is chiral. It is important to note that the device is planar, and current is injected via the flakes that are in contact with the metallic electrodes at the bottom. However, since the flake size is smaller than the contact gap, carriers need to transfer to the other overlapping flakes to complete the current path. Thus, along with the primarily planar component of charge flow, a small vertical “out-of-plane” component is present, as well. This is shown schematically in Figure 2c.

**Figure 3:**
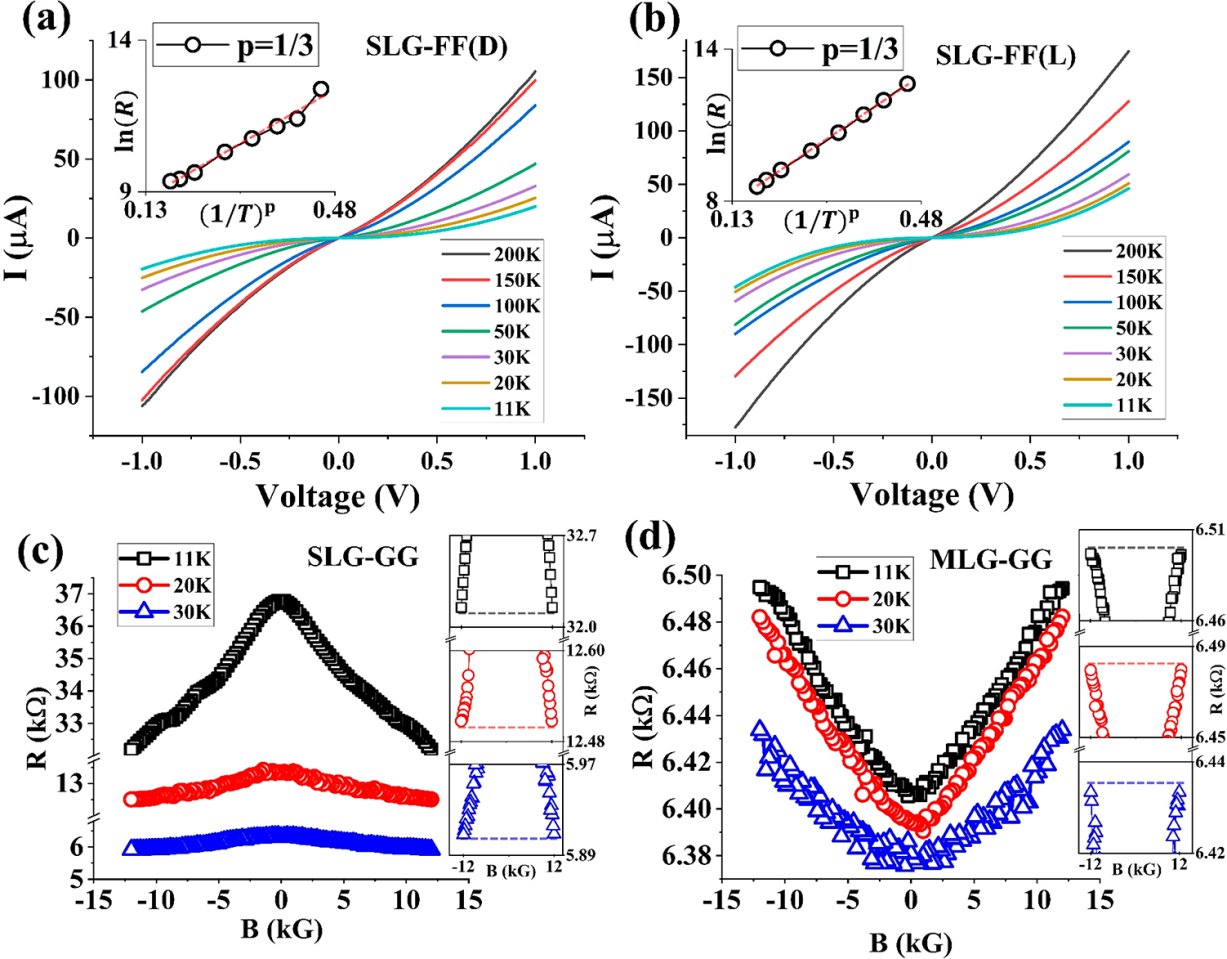
(a,b) Main image: Current–voltage (*I–V*) characterization of Fmoc-FF L/D functionalized SLG samples. Insets: Fitting with the VRH model. (c,d) Main image: MR characterization of SLG and MLG samples, respectively, functionalized with achiral Fmoc-GG molecules. The insets show the symmetry of the MR response. Resistance values are measured at 0.5 V.

Figure 3a,b show the temperature-dependent current–voltage (*I–V*) characteristics of the Fmoc-FF+SLG samples with both chiralities, measured at zero magnetic field. The *I–V* plots as well as the temperature-dependence of resistance of the Fmoc-GG+SLG/MLG samples are shown in the Supporting Information (Figure S3). The *I–V* characteristics have been found to be nonlinear relative to bias and semiconducting in terms of temperature dependence. Similar behavior has been reported in the literature for monolayer^49−51^ as well as multilayer rGO.^52^ In rGO, regions of highly conductive graphene islands with delocalized states are separated from each other via disordered regions or “barriers” with localized states, and conduction is limited by the temperature activated hopping mechanism through the localized states.^49,50,52^ In this case, the attachment of chiral molecules is also responsible for these barriers. As bias voltage is increased, electrons acquire sufficient energy and percolate through the flakes via multiple branches (field-assisted tunneling), which gives rise to the nonlinear bias-dependence. It is to be noted that in the present case, the graphene flakes are functionalized with chiral molecules, which is expected to induce CISS during transport.

The temperature-dependent *I–V* response in rGO or randomly distributed graphene islands is typically explained in terms of the variable-range hopping (VRH) model,^48−52^ in which resistance *R* scales with temperature *T* as follows:

*R* ∝ exp[(*T*_0_/*T*)^p^], where *T*_0_ is a parameter known as “characteristic temperature” and *p* is a fractional exponent equal to 1/(*d* + 1), where *d* is the dimensionality of the system. Figure 3a,b insets show logarithmic resistance (computed at 0.5 V) vs 1/*T*^1/3^. A linear dependence is found, which is consistent with the 2D structures of the rGO flakes.

The temperature dependence of resistance is weaker in MLG samples compared with the SLG samples (Figure S3). In the case of MLG, only the surface layer is functionalized, whereas the inner layers are not, and the transport occurs via both inner layers and the surface layers. The unfunctionalized inner layers offer fewer barriers and hence weaker temperature dependence of resistivity for the MLG samples.

Figure 3c shows the magnetoresistance (MR) of Fmoc-GG functionalized SLG flakes, contacted between Ni–Au electrodes, with a magnetic field in the out-of-plane direction. It is to be noted that the MR curve is symmetric, with *R*(+12 kG) = *R*(−12 kG), as shown in the insets. A negative MR, defined as *R*(0) – *R*(±12 kG)/*R*(±12 kG), of ∼15% is observed at 11 K for the Fmoc-GG+SLG samples, which gradually weakens with increasing temperature. As described above, the system is not a weakly disordered metallic system, and hence the standard weak-localization theory cannot be used to explain this negative MR. However, even in the VRH regime, such negative MR has been reported to appear, due to magnetic field driven carrier delocalization.^53^

Figure 3d shows the MR of Fmoc-GG functionalized MLG flakes. Unlike the SLG samples, a positive MR is observed with a magnitude of ∼12% at low temperatures. As before, the MR is symmetric, with *R*(+12 kG) = *R*(−12 kG), as shown in the insets. Thicker MLG layers are analogous to graphite, which is known to exhibit positive in-plane MR in the presence of an out-of-plane magnetic field.^54^ This is presumably because transport occurs via multiple layers, which offers significantly more carrier pathways than SLG. In each path, the carriers experience deflections due to magnetic field induced Lorentz force, which tends to deviate the carriers, resulting in a positive MR.

In general, MLG samples show lower resistance values compared with their SLG counterparts. We believe that the availability of multiple inner current paths via the inner layers of MLG causes lower resistance. The inner layers are not functionalized as discussed above, and hence, they offer fewer barriers, which lowers the overall resistance of the MLG samples.

**Figure 4:**
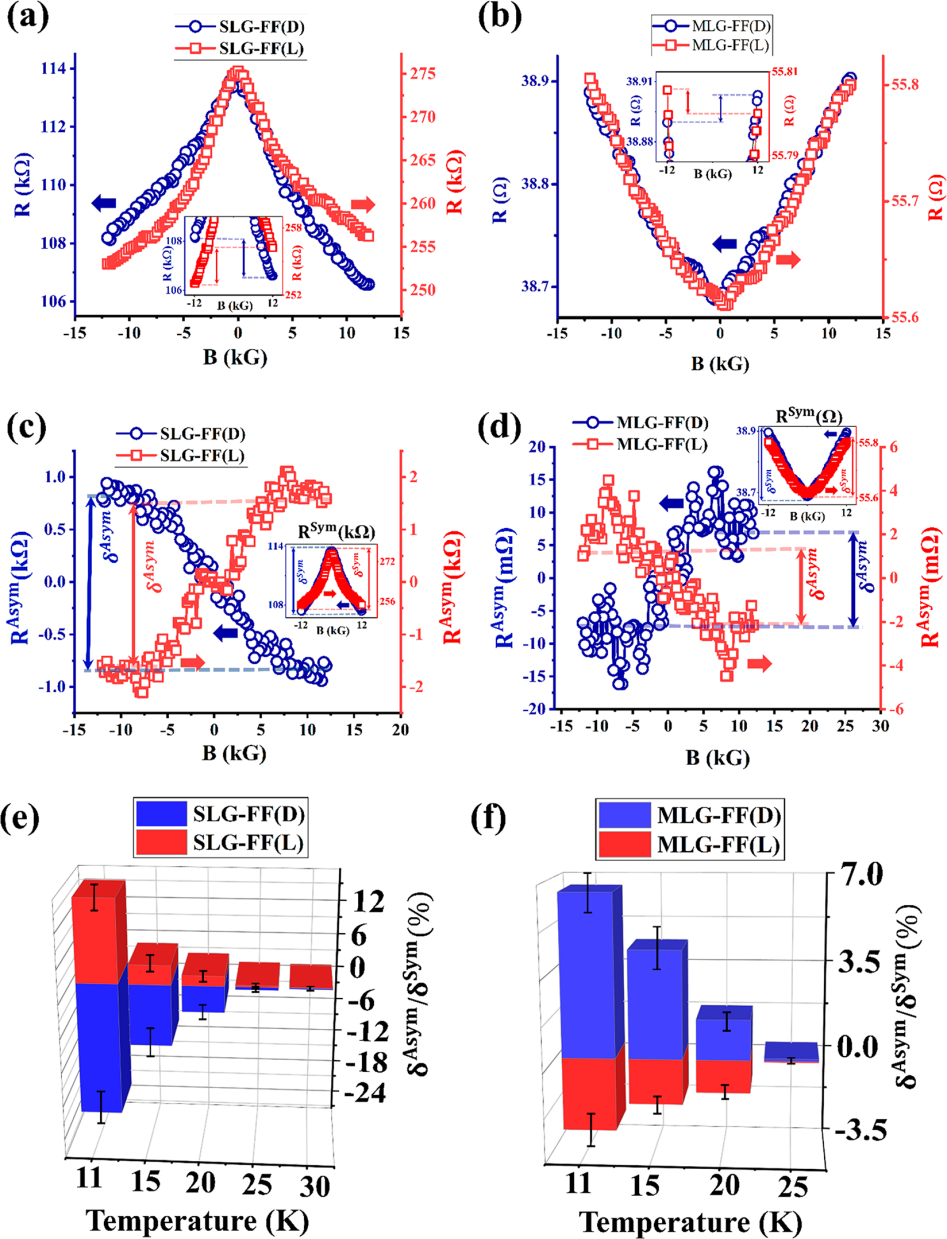
(a,b) Main images: MR responses of Fmoc-FF L/D functionalized SLG and MLG samples, respectively. The insets show the asymmetry of the MR responses. Resistance values are measured at 0.5 V. (c, d) Odd (main image) and even (insets) components of the MR response. (e, f) CISS signal as a function of temperature for both types of samples.

Figure 4a,b show the MR responses of chiral functionalized (using Fmoc-FF L/D) SLG and MLG samples, respectively. The background negative (positive) MR is present for SLG (MLG) samples. The new feature is an asymmetry in the MR response, which depends on the chirality, as clarified in the insets. For example, in the case of SLG samples (Figure 4a), *R*(+12 kG) _<_^>^*R* (−12 kG) for L and D chirality, respectively. For MLG samples, as shown in Figure 4b, this dependence is reversed, for example, *R*(+12 kG)_<_^>^*R* (−12 kG) for L and D chirality, respectively.

Such chirality-dependent MR asymmetry can be explained by invoking the standard CISS phenomenology.^2^ The electrons traveling through chiral (L/D) functionalized graphene flakes acquire a chirality-dependent spin polarization (up/down), which is either transmitted or blocked depending on the magnetization of the Ni spin detector, resulting in a chirality dependent asymmetry in the MR. Opposite chiralities induce opposite spin polarizations, and hence, the MR asymmetry is reversed for L and D chiralities. It is to be noted that in our experiments we have kept the magnetic field perpendicular to the sample plane and hence perpendicular to the planar current paths. This ensures that the results are minimally influenced by any electromagnetochiral effect.^55^ Observation of a CISS signal in such a transverse configuration also confirms the existence of transverse spin components perpendicular to the current path. This has been reported before in functionalized carbon nanotubes^13^ and is true for functionalized graphene as well. For ideal helical systems, the net transverse spin component is expected to be zero.^56^ However, in reality, the direction of spin depends on various factors such as electron energy or the details of the chiral medium and its coupling with the electrodes.^56^ These can result in nonzero transverse spin polarizations.

The opposite MR asymmetries of the SLG and MLG samples correlate with their respective chiralities, as discussed earlier in Figure 1b,c. The symmetric MR response from the Fmoc-GG functionalized samples shown in Figure 3c,d also correlates with the null CD result in Figure 1d. This is a clear indication that the supramolecular chirality associated with the peptide fibers is efficiently transferred to the conductive material.^12,14^ Even though the details about the mechanism of chiral induction are not fully known, it is worth noting that simple CD measurements of supramolecular aggregates gave predictable information about the asymmetry in the MR responses. Consequently, achiral fibers with null CD resulted in no spin selection. Although a correlation between the CISS effect and the sign and magnitude of the CD of the conductive material has been suggested in earlier reports,^57−59^ the present result shows that the CD signal of the chiral inductor could also infer the CISS response of the composite material.

Since the role of the chiral molecules is to introduce an asymmetry in the MR response, we isolate this contribution by computing the odd component of the MR function, defined as *R*^Asym^(*B*) = [*R*(*B*) – *R*(−*B*)]/2, shown in Figure 4c,d. Likewise, the CISS-independent background symmetric MR is quantified by the even component of the MR function: *R*^Sym^(*B*) = [*R*(*B*) + *R*(−*B*)]/2, as plotted in the insets of Figure 4c,d. The magnitude of this CISS-independent symmetric component is defined as δ^Sym^ = |*R*^Sym^(0 kG) – *R*^Sym^(±12 kG)|. Similarly, the strength of the CISS-dependent asymmetric component is computed as δ^Asym^ = *R*^Asym^(+ 12 kG) – *R*^Asym^(−12 kG), and the CISS signal is represented as δ^Asym^/δ^Sym^ × 100%.

Figure 4e and f summarize the δ^Asym^/δ^Sym^ responses for SLG and MLG samples, respectively, showing their temperature dependence. The CISS signal has been found to decrease with increasing temperature. While this is in contrast with the molecular systems, such temperature dependence is often observed in *solid-state* CISS devices.^60^ We also observe that the δ^Asym^/δ^Sym^ values of the MLG samples are generally lower than those of the SLG samples under the same conditions. This is expected, because, as discussed above, transport in MLG occurs via multiple inner layers, and these layers are less affected by the chiral molecules, which are attached on the surface layers. We measured four different SLG samples and nine different MLG samples. For the SLG samples, since the signal was stronger, we typically measured each device two or three times at a given temperature and bias. For the MLG samples, since the signal was weaker, we typically measured each device five or six times. The signals are reproducible, and the error bars shown in Figure 4e,f are derived from all of the above scans, i.e., including different devices as well as multiple scans on the same device. In Figure S4 (Supporting Information), we have added data obtained from a different set of samples, showing the reproducibility and variability of these results.

A self-consistent theoretical model of the CISS effect currently does not exist, and it is a subject of significant research activity.^1^ It is generally thought that spin–orbit interaction, along with spatial inversion asymmetry (due to the chiral structure) and time inversion asymmetry (due to the external magnetic field), are the necessary components for observation of this phenomenon.^1^ Spin–orbit interaction of pristine graphene is weak, due to the low atomic number of carbon,^61^ and hence the observation of the CISS effect discussed above is somewhat surprising. However, in the present case, there are several factors that may mitigate this effect:

(a) First, it has been reported by theoretical calculations that curved graphene layers have an additional spin–orbit term that arises due to the mixing of σ and π bands due to local curvature.^61^ Such curvature-induced spin–orbit interaction is estimated to be an order-of-magnitude stronger than the intrinsic spin–orbit coupling.^61^ In our case, on the molecular level, the chiral entities form a helical supramolecular structure. As discussed above, the chiral molecules are strongly attached to the graphene layers via π–π interactions. Hence, when these molecules self-assemble to form superhelical structures, so do the graphene layers attached to them (so-called “conformational chirality”), which makes the graphene layers curved, and this can enhance the spin–orbit coupling. The TEM images in Figure 1f,g and the FESEM image in Figure 2b indicate that the graphene layers are indeed curved. We also note that due to the relatively higher structural rigidity of MLG samples compared to SLG, such an effect is expected to be weaker in MLG, which is consistent with the observation of weaker CISS signals from MLG samples.

(b) Second, and perhaps more importantly, the system under consideration is rGO, instead of pristine graphene. Due to the synthesis mechanism of rGO, there exist impurity atoms (such as hydrogen) on the graphene lattice.^22,62^ Such impurities can cause local lattice distortion via sp^3^ hybridization, which can significantly enhance the local spin–orbit interaction.^63,64^ According to some studies, such enhanced spin–orbit interactions can approach values comparable to those in zinc-blende semiconductors.^63^ Such spin–orbit enhancements are presumably responsible for the CISS signal in rGO, even though it is not expected in pristine, impurity-free graphene. We note that such adatom-induced enhancement of the spin–orbit interaction is responsible for small spin relaxation times in graphene.^65^

Apart from the above factors that enhance the spin–orbit coupling in rGO, we note that there are several sources of magnetic defects, as well. The hydrogen adatoms discussed above can give rise to local magnetic moments.^66^ Carbon vacancies, which typically arise during GO synthesis and its thermal exfoliation reduction, are well-known magnetic defects.^22,62,67^ Such local magnetic interactions can also contribute to the observed CISS effect.

**Figure 5:**
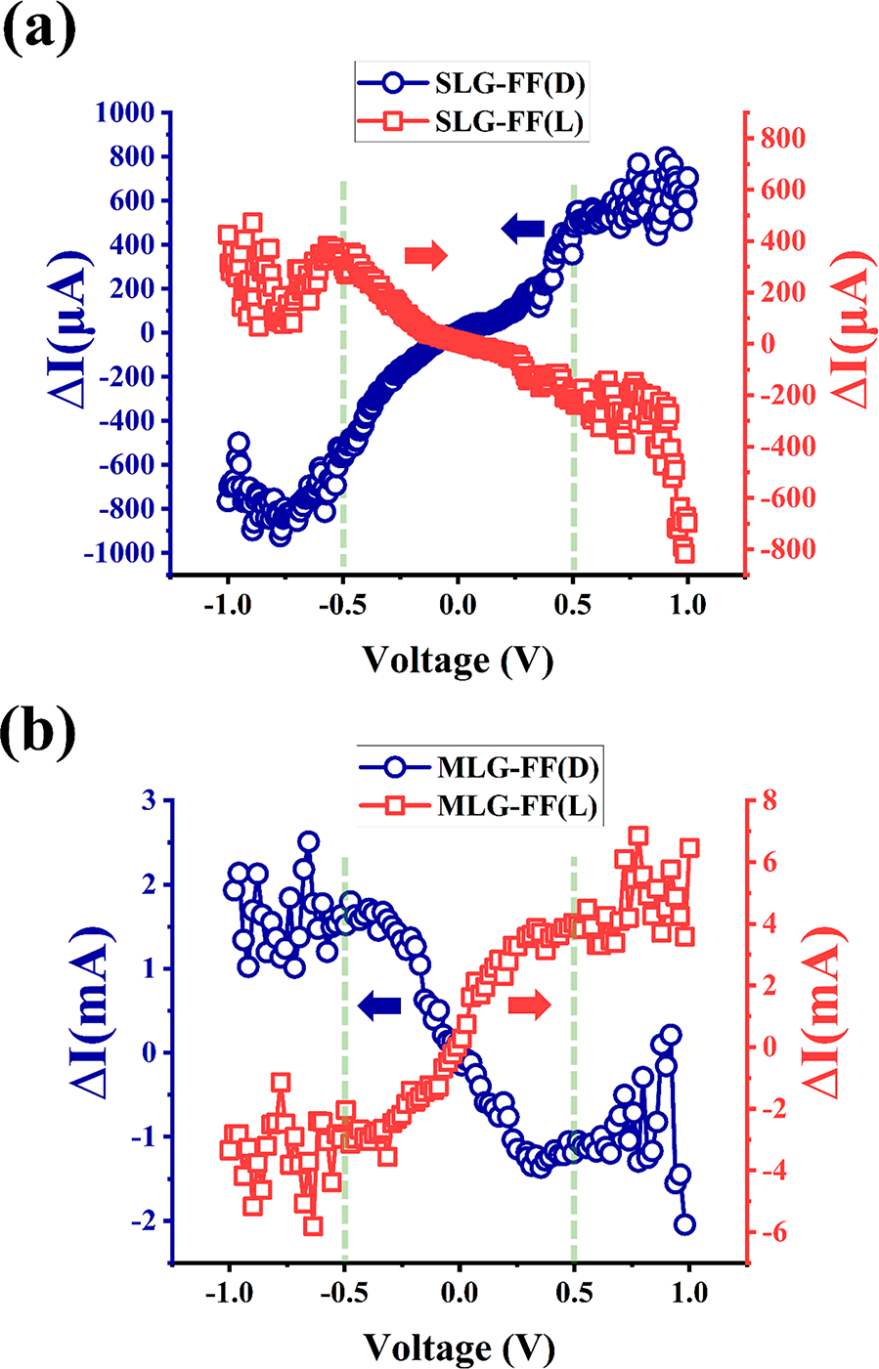
Bias dependence of Δ*I*, computed as *I*(+12 kG) – *I*(−12 kG), for Fmoc-FF L/D functionalized samples. The current difference approaches zero as the bias is reduced (approaching the linear range), consistent with Onsager’s reciprocity principle.

Figure 5 shows the differential current signal Δ*I*, computed as *I*(+12 kG) – *I*(−12 kG) as a function of the applied voltage bias. In a two-terminal measurement geometry with only one magnetic contact, as shown in Figure 2b and c, Δ*I* is expected to be zero according to Onsager’s reciprocity principle, at least in the linear region.^68^ As seen from Figure 5, Δ*I* approaches zero as voltage bias approaches zero, i.e., as the device enters the linear region of operation. The current differential Δ*I* due to the CISS effect increases with bias and manifests in the *nonlinear* region of transport in the present measurement geometry. This result is consistent with that observed recently in CNT based CISS devices.^13^ The CISS signal saturates around ∼0.5 V, and hence, the MR curves discussed in Figures 3 and 4 have been acquired at this optimum bias value.

## Conclusion

Graphene is a promising 2D material for emerging electronic and spintronic devices; however, its compatibility with the CISS effect was never explored before. In fact, weak spin–orbit interaction of graphene makes the occurrence of CISS unlikely in this material. Nevertheless, in this work, we demonstrated that the CISS effect manifests in rGO flakes functionalized by chiral dipeptide molecules. The chiral molecules organize in supramolecular structures, resulting in a “conformational chirality” of the attached graphene flakes. This introduces a curvature in the graphene flakes, as shown by the microscopic images discussed in this paper. Such curvatures as well as impurities in rGO can enhance the spin–orbit interaction and result in the CISS signal. The simple and straightforward methodology described in this work can be used to induce chirality and CISS properties in a multitude of easily accessible advanced 2D conductive materials, vastly expanding device design opportunities and associated applications. The observed CISS signal correlates with the supramolecular chirality of the medium, emphasizing the importance of “global chirality” as opposed to “local chirality” in the CISS phenomenon.^6,12,14^

## Materials and Methods

### Materials

The molecular structures of Fmoc-FF and Fmoc-GG are shown in Figure S1 (Supporting Information). As purchased SLG samples (Alfa Chemistry, CAS No.: 7782–42–5), prepared by thermal exfoliation reduction and hydrogen reduction of graphite oxide (GO), consist of one to five atomic layers of graphene with a typical flake size of ∼0.5–5μm. The oxygen content is estimated to be ∼7–7.5%. The MLG samples were procured from TCI Europe (product code: G0442, CAS No.: 1034343–98–0) and are 6–8 nm thick nanoplatelets with a width of ∼15 μm. No exfoliation step has been employed for the MLG samples; hence, it is expected that the chiral functionalization (described below) occurs only on the surface, whereas the inner layers remain impervious to it. This allows an investigation of the effect of sample thickness on the observed behavior.

N-Fluorenylmethoxycarbonyl-l-diphenylalanine (Fmoc-FF(L)) and N-fluorenylmethoxycarbonyl-d-diphenylalanine (Fmoc-FF (D)) were purchased from LifeTein, USA. N-Fluoroenylmethoxycarbonyl-diglycine (Fmoc-GG) was purchased from Fluorochem, UK. All Fmoc-peptides were used without further purification.

### Sample Preparation

To obtain the Fmoc-peptides + SLG/MLG composites, basic solutions of Fmoc-FF (L/D) and Fmoc-GG were first prepared. Fmoc-FF (L/D) and Fmoc-GG peptides were weighed separately into a vial, and deionized water was added to obtain a final concentration of 10 mM. The suspension was sonicated (HSt Powersonic 405 ultrasonic bath) for 1 h. Then, a NaOH solution (0.5 M) was added dropwise until a clear solution was obtained (pH = 10.7). The pH was measured using a HACH sensor PH 3 pH meter. The pH meter was calibrated using pH 4, pH 7, and pH 10 buffer solutions.

To prepare the graphene flake peptide solution (SLG or MLG), 0.7 mg of each graphene flake was separately weighed in a vial tube. The graphene flakes were suspended in 1 mL of a basic aqueous solution of Fmoc-FF (L/D) or Fmoc-GG (prepared above). The suspension was sonicated for 2.5 h in a cold ultrasonic bath and then centrifuged for 5 min at 10 000 rpm (Sigma 1–14 centrifuge). Finally, the supernatant was carefully collected.

Final hydrogels were obtained using GdL (glucono-δ-lactone) as a gelling agent for the Fmoc-FF + SLG/MLG composite solutions by adding 2 mol equiv of GdL and mixing by vortexing.^12^ Fmoc-GG + SLG/MLG hydrogels were obtained using Na_2_CO_3_ as a gelling agent by adding a final concentration of 25 mM sodium carbonate and mixed by vortexing.^11^

### Sample Characterization

The CD spectra were recorded using a Jasco J-815 spectropolarimeter with a xenon lamp of 150 W. The samples were measured into a 0.1 mm quartz cell (Hellma 0.1 mm quartz Suprasils), and the spectra were obtained from 200 to 350 nm with a 1 nm step and 1 s integration time per step at 20 °C. Measurements were performed with a 1:1 dilution ratio of peptide basic solutions or composite solutions in MiliQ water to keep sample absorbance below 2.

Raman spectra of commercial graphene flakes were collected on a Raman microscope NRS-5100 (JASCO, Japan) equipped with a Peltier cooled charge-coupled device (CCD) (1064 × 256 pixels) detector. The excitation line, provided by a diode laser emitting at 532 nm, was focused on the surface of the sample through a 100× objective lens. The spectral resolution was 2.1 cm^–1^, and each spectrum resulted from the average of three acquisitions, with 50 s of accumulation each. The samples were deposited on glass slides directly from the bottle and crushed to form a thin film.

For TEM, the samples were sonicated for 10 min and then drop cast onto a TEM Cu grid, coated with lacey carbon film. After 24 h drying in a normal atmosphere, the grid was mounted on the JEM-ARM200cf S/TEM, to obtain the TEM images.

## Supporting Information Available

Molecular structures, HT spectra, additional current–voltage characterizations, additional MR data from a different set of samples (PDF)

## References

[ref1] Evers F.; Aharony A.; Bar-Gill N.; Entin-Wohlman O.; Hedegård P.; Hod O.; Jelinek P.; Kamieniarz G.; Lemeshko M.; Michaeli K.; Mujica V.; Naaman R.; Paltiel Y.; Refaely-Abramson S.; Tal O.; Thijssen J.; Thoss M.; van Ruitenbeek J. M.; Venkataraman L.; Waldeck D. H.; Yan B.; Kronik L. Theory of Chirality Induced Spin Selectivity: Progress and Challenges. Adv. Mater. 2022, 34 (13), 2106629 10.1002/adma.202106629. 35064943

[ref2] Aiello C. D.; Abendroth J. M.; Abbas M.; Afanasev A.; Agarwal S.; Banerjee A. S.; Beratan D. N.; Belling J. N.; Berche B.; Botana A.; Caram J. R.; Celardo G. L.; Cuniberti G.; Garcia-Etxarri A.; Dianat A.; Diez-Perez I.; Guo Y.; Gutierrez R.; Herrmann C.; Hihath J.; Kale S.; Kurian P.; Lai Y.-C.; Liu T.; Lopez A.; Medina E.; Mujica V.; Naaman R.; Noormandipour M.; Palma J. L.; Paltiel Y.; Petuskey W.; Ribeiro-Silva J. C.; Saenz J. J.; Santos E. J. G.; Solyanik-Gorgone M.; Sorger V. J.; Stemer D. M.; Ugalde J. M.; Valdes-Curiel A.; Varela S.; Waldeck D. H.; Wasielewski M. R.; Weiss P. S.; Zacharias H.; Wang Q. H. A Chirality-Based Quantum Leap. ACS Nano 2022, 16 (4), 4989–5035. 10.1021/acsnano.1c01347. 35318848 PMC9278663

[ref3] Gohler B.; Hamelbeck V.; Markus T. Z.; Kettner M.; Hanne G. F.; Vager Z.; Naaman R.; Zacharias H. Spin Selectivity in Electron Transmission Through Self-Assembled Monolayers of Double-Stranded DNA. Science 2011, 331 (6019), 894–897. 10.1126/science.1199339. 21330541

[ref4] Xie Z.; Markus T. Z.; Cohen S. R.; Vager Z.; Gutierrez R.; Naaman R. Spin Specific Electron Conduction through DNA Oligomers. Nano Lett. 2011, 11 (11), 4652–4655. 10.1021/nl2021637. 21961931

[ref5] Kiran V.; Mathew S. P.; Cohen S. R.; Hernandez Delgado I.; Lacour J.; Naaman R. Helicenes—A New Class of Organic Spin Filter. Adv. Mater. 2016, 28 (10), 1957–1962. 10.1002/adma.201504725. 26742997

[ref6] Kulkarni C.; Mondal A. K.; Das T. K.; Grinbom G.; Tassinari F.; Mabesoone M. F. J.; Meijer E. W.; Naaman R. Highly Efficient and Tunable Filtering of Electrons’ Spin by Supramolecular Chirality of Nanofiber-Based Materials. Adv. Mater. 2020, 32 (7), 1904965 10.1002/adma.201904965. 31922628

[ref7] Alam K. M.; Pramanik S. Spin Filtering through Single-Wall Carbon Nanotubes Functionalized with Single-Stranded DNA. Adv. Funct. Mater. 2015, 25 (21), 3210–3218. 10.1002/adfm.201500494.

[ref8] Alam K. M.; Pramanik S. Spin Filtering with Poly-T Wrapped Single Wall Carbon Nanotubes. Nanoscale 2017, 9 (16), 5155–5163. 10.1039/C6NR09395G. 28393942

[ref9] Rahman Md. W.; Alam K. M.; Pramanik S. Long Carbon Nanotubes Functionalized with DNA and Implications for Spintronics. ACS Omega 2018, 3 (12), 17108–17115. 10.1021/acsomega.8b02237. 31458331 PMC6644094

[ref10] Rahman Md. W.; Firouzeh S.; Mujica V.; Pramanik S. Carrier Transport Engineering in Carbon Nanotubes by Chirality-Induced Spin Polarization. ACS Nano 2020, 14 (3), 3389–3396. 10.1021/acsnano.9b09267. 32096973

[ref11] Rahman Md. W.; Mañas-Torres M. C.; Firouzeh S.; Cuerva J. M.; Álvarez de Cienfuegos L.; Pramanik S. Molecular Functionalization and Emergence of Long-Range Spin-Dependent Phenomena in Two-Dimensional Carbon Nanotube Networks. ACS Nano 2021, 15 (12), 20056–20066. 10.1021/acsnano.1c07739. 34870421

[ref12] Rahman M. W.; Manas-Torres M. C.; Firouzeh S.; Illescas-Lopez S.; Cuerva J. M.; Lopez-Lopez M. T.; de Cienfuegos L. A.; Pramanik S. Chirality-Induced Spin Selectivity in Heterochiral Short-Peptide-Carbon-Nanotube Hybrid Networks: Role of Supramolecular Chirality. ACS Nano 2022, 16 (10), 16941–16953. 10.1021/acsnano.2c07040. 36219724

[ref13] Hossain M. A.; Illescas-Lopez S.; Nair R.; Cuerva J. M.; Alvarez de Cienfuegos L.; Pramanik S. Transverse Magnetoconductance in Two-Terminal Chiral Spin-Selective Devices. Nanoscale Horiz. 2023, 8 (3), 320–330. 10.1039/D2NH00502F. 36740957

[ref14] Firouzeh S.; Illescas-Lopez S.; Hossain M. A.; Cuerva J. M.; Álvarez de Cienfuegos L.; Pramanik S. Chirality-Induced Spin Selectivity in Functionalized Carbon Nanotube Networks: The Role of Spin-Orbit Coupling. J. Chem. Phys. 2023, 159 (3), 034708 10.1063/5.0156348. 37466230

[ref15] Inui A.; Aoki R.; Nishiue Y.; Shiota K.; Kousaka Y.; Shishido H.; Hirobe D.; Suda M.; Ohe J.; Kishine J.; Yamamoto H. M.; Togawa Y. Chirality-Induced Spin-Polarized State of a Chiral Crystal CrNb3S6. Phys. Rev. Lett. 2020, 124 (16), 166602 10.1103/PhysRevLett.124.166602. 32383920

[ref16] Shiota K.; Inui A.; Hosaka Y.; Amano R.; O̅nuki Y.; Hedo M.; Nakama T.; Hirobe D.; Ohe J.; Kishine J.; Yamamoto H. M.; Shishido H.; Togawa Y. Chirality-Induced Spin Polarization over Macroscopic Distances in Chiral Disilicide Crystals. Phys. Rev. Lett. 2021, 127 (12), 126602 10.1103/PhysRevLett.127.126602. 34597079

[ref17] Kim Y.-H.; Zhai Y.; Lu H.; Pan X.; Xiao C.; Gaulding E. A.; Harvey S. P.; Berry J. J.; Vardeny Z. V.; Luther J. M.; Beard M. C. Chiral-Induced Spin Selectivity Enables a Room-Temperature Spin Light-Emitting Diode. Science 2021, 371 (6534), 1129–1133. 10.1126/science.abf5291. 33707260

[ref18] Lu H.; Wang J.; Xiao C.; Pan X.; Chen X.; Brunecky R.; Berry J. J.; Zhu K.; Beard M. C.; Vardeny Z. V. Spin-Dependent Charge Transport through 2D Chiral Hybrid Lead-Iodide Perovskites. Sci. Adv. 2019, 5 (12), eaay0571 10.1126/sciadv.aay0571. 31840072 PMC6897542

[ref19] Geim A. K. Graphene: Status and Prospects. Science 2009, 324 (5934), 1530–1534. 10.1126/science.1158877. 19541989

[ref20] Novoselov K. S.; Jiang Z.; Zhang Y.; Morozov S. V.; Stormer H. L.; Zeitler U.; Maan J. C.; Boebinger G. S.; Kim P.; Geim A. K. Room-Temperature Quantum Hall Effect in Graphene. Science 2007, 315 (5817), 1379–1379. 10.1126/science.1137201. 17303717

[ref21] Tombros N.; Jozsa C.; Popinciuc M.; Jonkman H. T.; van Wees B. J. Electronic Spin Transport and Spin Precession in Single Graphene Layers at Room Temperature. Nature 2007, 448 (7153), 571–574. 10.1038/nature06037. 17632544

[ref22] Han W.; Kawakami R. K.; Gmitra M.; Fabian J. Graphene Spintronics. Nat. Nanotechnol. 2014, 9 (10), 794–807. 10.1038/nnano.2014.214. 25286274

[ref23] Qing G.; Zhao S.; Xiong Y.; Lv Z.; Jiang F.; Liu Y.; Chen H.; Zhang M.; Sun T. Chiral Effect at Protein/Graphene Interface: A Bioinspired Perspective To Understand Amyloid Formation. J. Am. Chem. Soc. 2014, 136 (30), 10736–10742. 10.1021/ja5049626. 25011035

[ref24] Guo L.; Zhang Q.; Huang Y.; Han Q.; Wang Y.; Fu Y. The Application of Thionine-Graphene Nanocomposite in Chiral Sensing for Tryptophan Enantiomers. Bioelectrochemistry 2013, 94, 87–93. 10.1016/j.bioelechem.2013.09.002. 24084594

[ref25] Cheng Q.; Pei H.; Ma Q.; Guo R.; Liu N.; Mo Z. Chiral Graphene Materials for Enantiomer Separation. Chem. Eng. J. 2023, 452, 139499 10.1016/j.cej.2022.139499.

[ref26] Zhao B.; Yang S.; Deng J.; Pan K. Chiral Graphene Hybrid Materials: Structures, Properties, and Chiral Applications. Adv. Sci. 2021, 8 (7), 2003681 10.1002/advs.202003681. PMC8025009 33854894

[ref27] Azlouk M.; Durmaz M.; Zor E.; Bingol H. Graphene-Based Recyclable and Bifunctional Heterogeneous Chiral Catalyst for Direct Asymmetric Aldol Reaction. Mater. Chem. Phys. 2020, 239, 122298 10.1016/j.matchemphys.2019.122298.

[ref28] Mao X.; Zhao H.; Luo L.; Tian D.; Li H. Highly Sensitive Chiral Recognition of Amino Propanol in Serum with R-Mandelic Acid-Linked Calix[4]Arene Modified Graphene. J. Mater. Chem. C 2015, 3 (6), 1325–1329. 10.1039/C4TC02365J.

[ref29] Huang H.; Hu L.; Sun Y.; Liu Y.; Kang Z.; MacFarlane D. R. Preparation of Chiral Graphene Oxides by Covalent Attachment of Chiral Cysteines for Voltammetric Recognition of Tartrates. Microchim. Acta 2019, 186 (5), 298 10.1007/s00604-019-3415-8. 31025191

[ref30] Shang X.; Park C. H.; Jung G. Y.; Kwak S. K.; Oh J. H. Highly Enantioselective Graphene-Based Chemical Sensors Prepared by Chiral Noncovalent Functionalization. ACS Appl. Mater. Interfaces 2018, 10 (42), 36194–36201. 10.1021/acsami.8b13517. 30270614

[ref31] Suzuki N.; Wang Y.; Elvati P.; Qu Z.-B.; Kim K.; Jiang S.; Baumeister E.; Lee J.; Yeom B.; Bahng J. H.; Lee J.; Violi A.; Kotov N. A. Chiral Graphene Quantum Dots. ACS Nano 2016, 10 (2), 1744–1755. 10.1021/acsnano.5b06369. 26743467

[ref32] Furlan de Oliveira R.; Livio P. A.; Montes-García V.; Ippolito S.; Eredia M.; Fanjul-Bolado P.; González García M. B.; Casalini S.; Samorì P. Liquid-Gated Transistors Based on Reduced Graphene Oxide for Flexible and Wearable Electronics. Adv. Funct. Mater. 2019, 29 (46), 1905375 10.1002/adfm.201905375.

[ref33] Fowler J. D.; Allen M. J.; Tung V. C.; Yang Y.; Kaner R. B.; Weiller B. H. Practical Chemical Sensors from Chemically Derived Graphene. ACS Nano 2009, 3 (2), 301–306. 10.1021/nn800593m. 19236064

[ref34] Eda G.; Fanchini G.; Chhowalla M. Large-Area Ultrathin Films of Reduced Graphene Oxide as a Transparent and Flexible Electronic Material. Nat. Nanotechnol. 2008, 3 (5), 270–274. 10.1038/nnano.2008.83. 18654522

[ref35] Zhang Y.; Gu H.; Yang Z.; Xu B. Supramolecular Hydrogels Respond to Ligand-Receptor Interaction. J. Am. Chem. Soc. 2003, 125 (45), 13680–13681. 10.1021/ja036817k. 14599204

[ref36] Jayawarna V.; Ali M.; Jowitt T. A.; Miller A. F.; Saiani A.; Gough J. E.; Ulijn R. V. Nanostructured Hydrogels for Three-Dimensional Cell Culture Through Self-Assembly of Fluorenylmethoxycarbonyl-Dipeptides. Adv. Mater. 2006, 18 (5), 611–614. 10.1002/adma.200501522.

[ref37] Gila-Vilchez C.; Mañas-Torres M. C.; González-Vera J. A.; Franco-Montalban F.; Tamayo J. A.; Conejero-Lara F.; Cuerva J. M.; Lopez-Lopez M. T.; Orte A.; Álvarez de Cienfuegos L. Insights into the Co-Assemblies Formed by Different Aromatic Short-Peptide Amphiphiles. Polym. Chem. 2021, 12 (47), 6832–6845. 10.1039/D1PY01366A.

[ref38] Mañas-Torres M. C.; Gila-Vilchez C. A.; González-Vera J.; Conejero-Lara F.; Blanco V.; Cuerva J. M.; Lopez-Lopez M.; Orte A.; Álvarez de Cienfuegos L. In Situ Real-Time Monitoring of the Mechanism of Self-Assembly of Short Peptide Supramolecular Polymers. Mater. Chem. Front. 2021, 5 (14), 5452–5462. 10.1039/D1QM00477H.

[ref39] Mañas-Torres M. C.; Gila-Vilchez C.; Vazquez-Perez F. J.; Kuzhir P.; Momier D.; Scimeca J.-C.; Borderie A.; Goracci M.; Burel-Vandenbos F.; Blanco-Elices C.; Rodriguez I. A.; Alaminos M.; Álvarez de Cienfuegos L.; Lopez-Lopez M. T. Injectable Magnetic-Responsive Short-Peptide Supramolecular Hydrogels: Ex Vivo and In Vivo Evaluation. ACS Appl. Mater. Interfaces 2021, 13 (42), 49692–49704. 10.1021/acsami.1c13972. 34645258 PMC8554763

[ref40] Contreras-Montoya R.; Bonhome-Espinosa A. B.; Orte A.; Miguel D.; Delgado-López J. M.; Duran J. D. G.; Cuerva J. M.; Lopez-Lopez M. T.; Álvarez de Cienfuegos L. Iron Nanoparticles-Based Supramolecular Hydrogels to Originate Anisotropic Hybrid Materials with Enhanced Mechanical Strength. Mater. Chem. Front. 2018, 2 (4), 686–699. 10.1039/C7QM00573C.

[ref41] Illescas-Lopez S.; Martin-Romera J. D.; Mañas-Torres M. C.; Lopez-Lopez M. T.; Cuerva J. M.; Gavira J. A.; Carmona F. J.; Álvarez de Cienfuegos L. Short-Peptide Supramolecular Hydrogels for In Situ Growth of Metal-Organic Framework-Peptide Biocomposites. ACS Appl. Mater. Interfaces 2023, 15 (27), 32597–32609. 10.1021/acsami.3c06943. 37390355 PMC10347120

[ref42] Fichman G.; Adler-Abramovich L.; Manohar S.; Mironi-Harpaz I.; Guterman T.; Seliktar D.; Messersmith P. B.; Gazit E. Seamless Metallic Coating and Surface Adhesion of Self-Assembled Bioinspired Nanostructures Based on Di-(3,4-Dihydroxy-l-Phenylalanine) Peptide Motif. ACS Nano 2014, 8 (7), 7220–7228. 10.1021/nn502240r. 24936704 PMC4108209

[ref43] Mañas-Torres M. C.; Ramírez-Rodríguez G. B.; García-Peiro J. I.; Parra-Torrejón B.; Cuerva J. M.; Lopez-Lopez M. T.; Álvarez de Cienfuegos L.; Delgado-López J. M. Organic/Inorganic Hydrogels by Simultaneous Self-Assembly and Mineralization of Aromatic Short-Peptides. Inorg. Chem. Front. 2022, 9 (4), 743–752. 10.1039/D1QI01249E.

[ref44] Malard L. M.; Pimenta M. A.; Dresselhaus G.; Dresselhaus M. S. Raman Spectroscopy in Graphene. Phys. Rep. 2009, 473 (5–6), 51–87. 10.1016/j.physrep.2009.02.003.

[ref45] Wang X. Y.; Miao L.; Liu C. Y.; Gao J.; Chen Y. Thermoelectric Enhancement of Polyaniline Grafting from Graphene Oxide. Mater. Sci. Forum 2016, 847, 153–160. 10.4028/www.scientific.net/MSF.847.153.

[ref46] Zhang K.; Fischer S.; Geissler A.; Brendler E. Analysis of Carboxylate Groups in Oxidized Never-Dried Cellulose II Catalyzed by TEMPO and 4-Acetamide-TEMPO. Carbohydr. Polym. 2012, 87 (1), 894–900. 10.1016/j.carbpol.2011.08.090. 34663051

[ref47] Abid; Sehrawat P.; Islam S. S.; Mishra P.; Ahmad S. Reduced Graphene Oxide (RGO) Based Wideband Optical Sensor and the Role of Temperature, Defect States and Quantum Efficiency. Sci. Rep. 2018, 8 (1), 3537 10.1038/s41598-018-21686-2. 29476091 PMC5824820

[ref48] Iacovella F.; Trinsoutrot P.; Mitioglu A.; Conédéra V.; Pierre M.; Raquet B.; Goiran M.; Vergnes H.; Caussat B.; Plochocka P.; Escoffier W. Magneto-Transport Properties of a Random Distribution of Few-Layer Graphene Patches. J. Appl. Phys. 2014, 116 (19), 193705 10.1063/1.4901953.

[ref49] Kaiser A. B.; Gómez-Navarro C.; Sundaram R. S.; Burghard M.; Kern K. Electrical Conduction Mechanism in Chemically Derived Graphene Monolayers. Nano Lett. 2009, 9 (5), 1787–1792. 10.1021/nl803698b. 19331348

[ref50] Gómez-Navarro C.; Weitz R. T.; Bittner A. M.; Scolari M.; Mews A.; Burghard M.; Kern K. Electronic Transport Properties of Individual Chemically Reduced Graphene Oxide Sheets. Nano Lett. 2007, 7 (11), 3499–3503. 10.1021/nl072090c. 17944526

[ref51] Eda G.; Mattevi C.; Yamaguchi H.; Kim H.; Chhowalla M. Insulator to Semimetal Transition in Graphene Oxide. J. Phys. Chem. C 2009, 113 (35), 15768–15771. 10.1021/jp9051402.

[ref52] Joung D.; Zhai L.; Khondaker S. I. Coulomb Blockade and Hopping Conduction in Graphene Quantum Dots Array. Phys. Rev. B 2011, 83 (11), 115323 10.1103/PhysRevB.83.115323.

[ref53] Pichard J.-L.; Sanquer M.; Slevin K.; Debray P. Broken Symmetries and Localization Lengths in Anderson Insulators: Theory and Experiment. Phys. Rev. Lett. 1990, 65 (14), 1812–1815. 10.1103/PhysRevLett.65.1812. 10042368

[ref54] Kempa H.; Semmelhack H. C.; Esquinazi P.; Kopelevich Y. Absence of Metal-Insulator Transition and Coherent Interlayer Transport in Oriented Graphite in Parallel Magnetic Fields. Solid State Commun. 2003, 125 (1), 1–5. 10.1016/S0038-1098(02)00711-1.

[ref55] Rikken G. L. J. A.; Fölling J.; Wyder P. Electrical Magnetochiral Anisotropy. Phys. Rev. Lett. 2001, 87 (23), 236602 10.1103/PhysRevLett.87.236602. 11736466

[ref56] Dalum S.; Hedegård P. Theory of Chiral Induced Spin Selectivity. Nano Lett. 2019, 19 (8), 5253–5259. 10.1021/acs.nanolett.9b01707. 31265313

[ref57] Bloom B. P.; Graff B. M.; Ghosh S.; Beratan D. N.; Waldeck D. H. Chirality Control of Electron Transfer in Quantum Dot Assemblies. J. Am. Chem. Soc. 2017, 139 (26), 9038–9043. 10.1021/jacs.7b04639. 28609095

[ref58] Ortuño A. M.; Reiné P.; Resa S.; Álvarez de Cienfuegos L.; Blanco V.; Paredes J. M.; Mota A. J.; Mazzeo G.; Abbate S.; Ugalde J. M.; Mujica V.; Longhi G.; Miguel D.; Cuerva J. M. Extended Enantiopure Ortho-Phenylene Ethylene (o-OPE)-Based Helical Systems as Scaffolds for Supramolecular Architectures: A Study of Chiroptical Response and Its Connection to the CISS Effect. Org. Chem. Front. 2021, 8 (18), 5071–5086. 10.1039/D1QO00822F.

[ref59] Ortuño A. M.; Reiné P.; Álvarez de Cienfuegos L.; Márquez I. R.; Dednam W.; Lombardi E. B.; Palacios J. J.; Leary E.; Longhi G.; Mujica V.; Millán A.; González M. T.; Zotti L. A.; Miguel D.; Cuerva J. M. Chiral Single-Molecule Potentiometers Based on Stapled Ortho- Oligo(Phenylene)Ethynylenes. Angew. Chem., Int. Ed. 2023, 62 (16), e202218640 10.1002/anie.202218640. 36806838

[ref60] Liu T.; Wang X.; Wang H.; Shi G.; Gao F.; Feng H.; Deng H.; Hu L.; Lochner E.; Schlottmann P.; von Molnár S.; Li Y.; Zhao J.; Xiong P. Linear and Nonlinear Two-Terminal Spin-Valve Effect from Chirality-Induced Spin Selectivity. ACS Nano 2020, 14 (11), 15983–15991. 10.1021/acsnano.0c07438. 33136367

[ref61] Huertas-Hernando D.; Guinea F.; Brataas A. Spin-Orbit Coupling in Curved Graphene, Fullerenes, Nanotubes, and Nanotube Caps. Phys. Rev. B 2006, 74 (15), 155426 10.1103/PhysRevB.74.155426.

[ref62] Augustyniak-Jabłokow M. A.; Carmieli R.; Strzelczyk R.; Fedaruk R.; Tadyszak K. Electron Spin Echo Studies of Hydrothermally Reduced Graphene Oxide. J. Phys. Chem. C 2021, 125 (7), 4102–4109. 10.1021/acs.jpcc.0c11316.

[ref63] Castro Neto A. H.; Guinea F. Impurity-Induced Spin-Orbit Coupling in Graphene. Phys. Rev. Lett. 2009, 103 (2), 026804 10.1103/PhysRevLett.103.026804. 19659232

[ref64] Gmitra M.; Kochan D.; Fabian J. Spin-Orbit Coupling in Hydrogenated Graphene. Phys. Rev. Lett. 2013, 110 (24), 246602 10.1103/PhysRevLett.110.246602. 25165949

[ref65] Ertler C.; Konschuh S.; Gmitra M.; Fabian J. Electron Spin Relaxation in Graphene: The Role of the Substrate. Phys. Rev. B 2009, 80 (4), 041405 10.1103/PhysRevB.80.041405.

[ref66] Sofo J. O.; Usaj G.; Cornaglia P. S.; Suarez A. M.; Hernández-Nieves A. D.; Balseiro C. A. Magnetic Structure of Hydrogen-Induced Defects on Graphene. Phys. Rev. B 2012, 85 (11), 115405 10.1103/PhysRevB.85.115405.

[ref67] Feicht P.; Eigler S. Defects in Graphene Oxide as Structural Motifs. ChemNanoMat 2018, 4 (3), 244–252. 10.1002/cnma.201700357.

[ref68] Yang X.; van der Wal C. H.; van Wees B. J. Spin-Dependent Electron Transmission Model for Chiral Molecules in Mesoscopic Devices. Phys. Rev. B 2019, 99 (2), 024418 10.1103/PhysRevB.99.024418.

