# Supplementary material for: Chirality-Induced Spin Selectivity in Supramolecular Chirally Functionalized Graphene
Source: ACS Nano. 2023 Sep 5;17(20):20424–33. doi: 10.1021/acsnano.3c06903 (PMC10604086; doi:10.1021/acsnano.3c06903)
Supplement: Supplementary file 1 — nn3c06903_si_001.pdf [file nn3c06903_si_001.pdf]

Supporting Information

Chirality-induced Spin Selectivity in Supramolecular Chirally Functionalized Graphene

Seyedamin Firouzeh,[a] Sara Illescas-Lopez,[b] Md Anik Hossain,[a] Juan Manuel Cuerva,[b] Luis Álvarez de Cienfuegos,[b,c,*] Sandipan Pramanik[a,*]

[a] Department of Electrical and Computer Engineering, University of Alberta, Edmonton, Alberta T6G 1H9, Canada

[b] Universidad de Granada, Departamento de Química Orgánica, Unidad de Excelencia Química Aplicada a Biomedicina y Medioambiente, C. U. Fuentenueva, Avda. Severo Ochoa s/n, E-18071 Granada, Spain

[c] Instituto de Investigación Biosanitaria ibs. Avda. De Madrid, 15, E-18016 Granada, Spain

*Corresponding Authors: lac@ugr.es, spramani@ualberta.ca

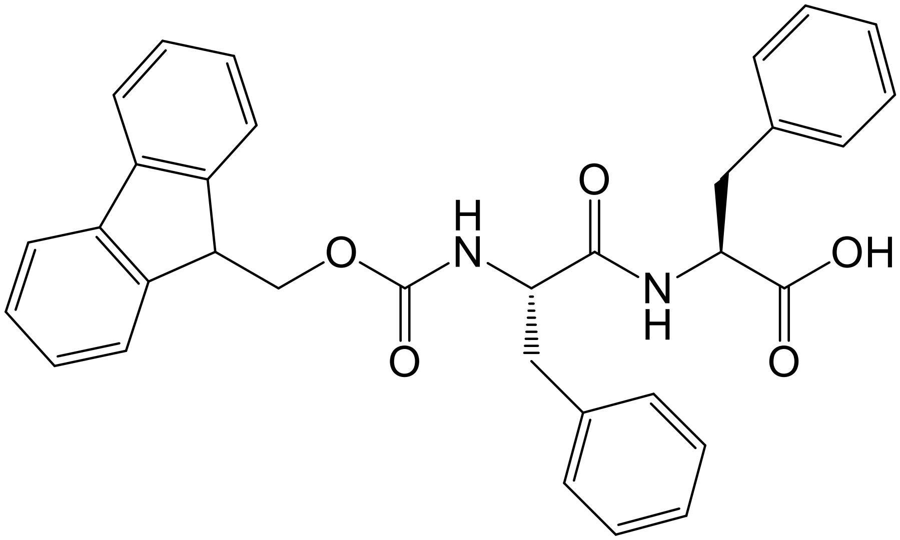

Fmoc-FF (L)

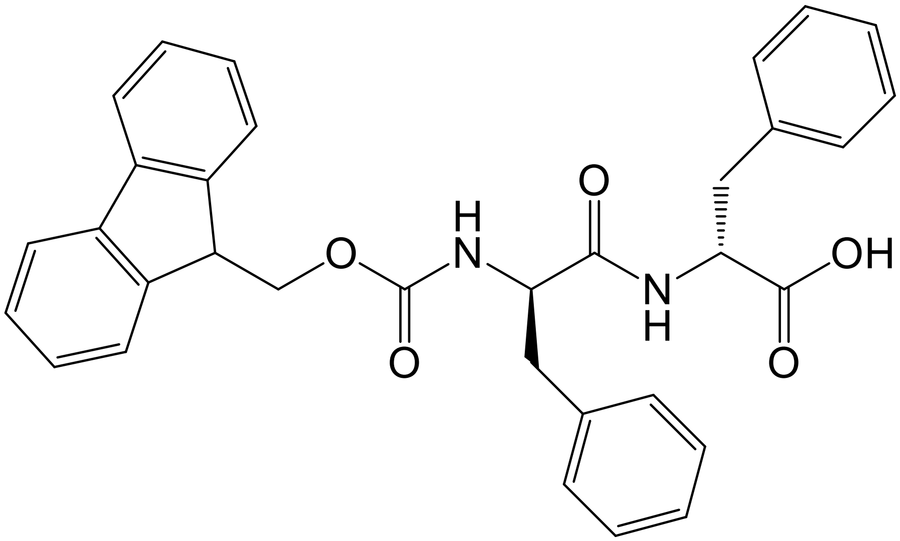

Fmoc-FF (D)

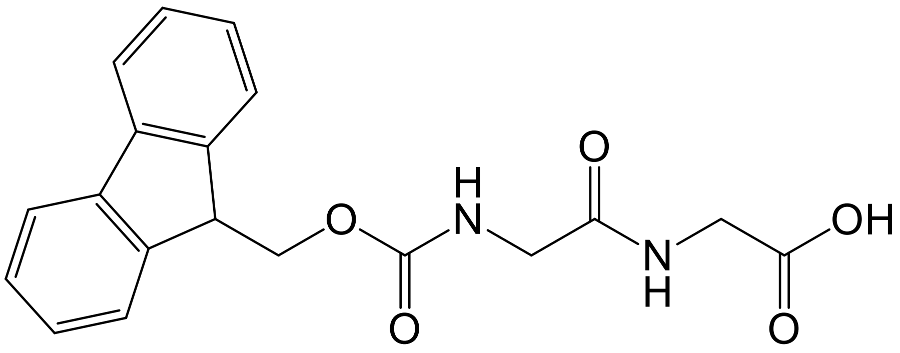

Fmoc-GG

Figure S1. Molecular structures of Fmoc-FF (L), Fmoc-FF (D) and Fmoc-GG.

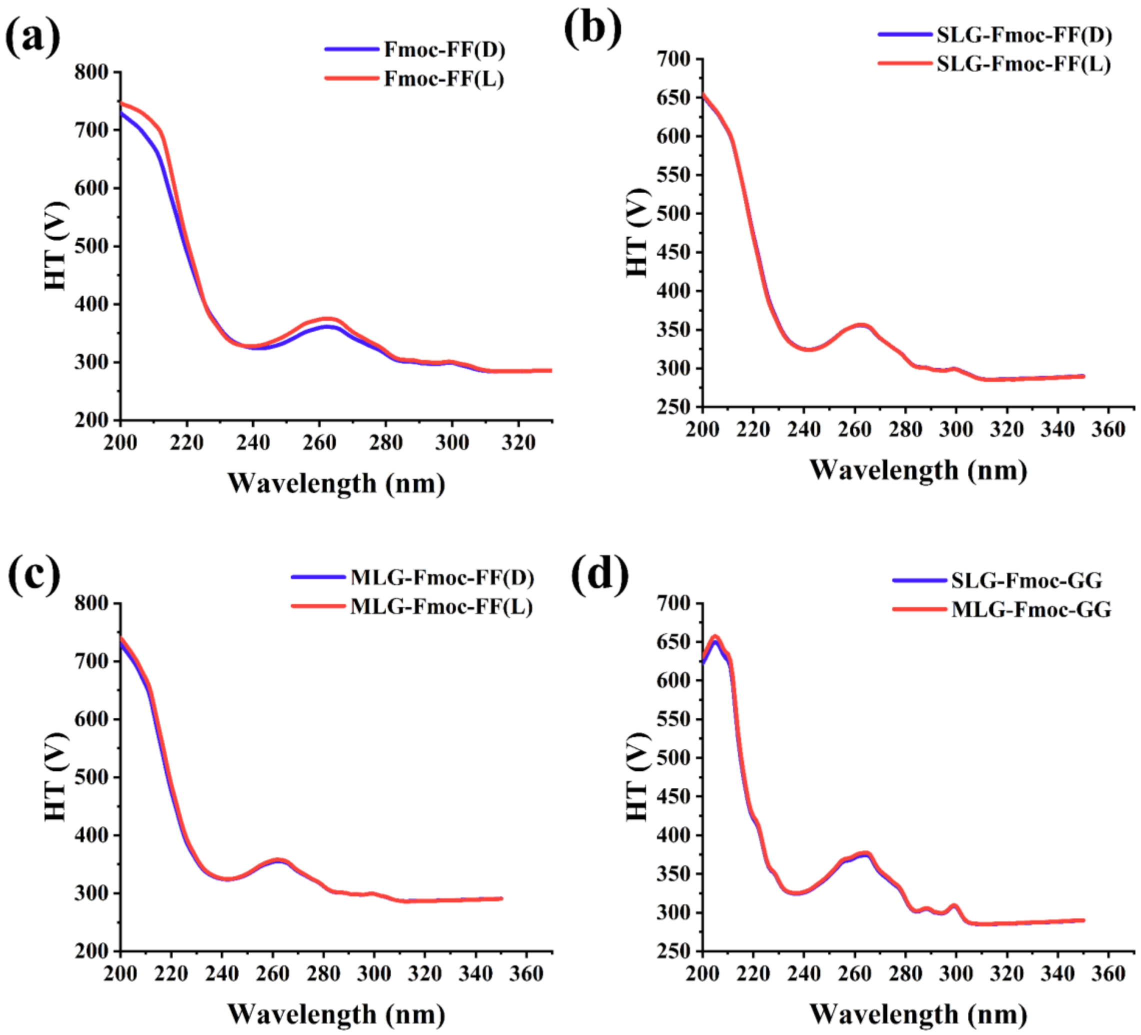

Figure S2. HT spectra.

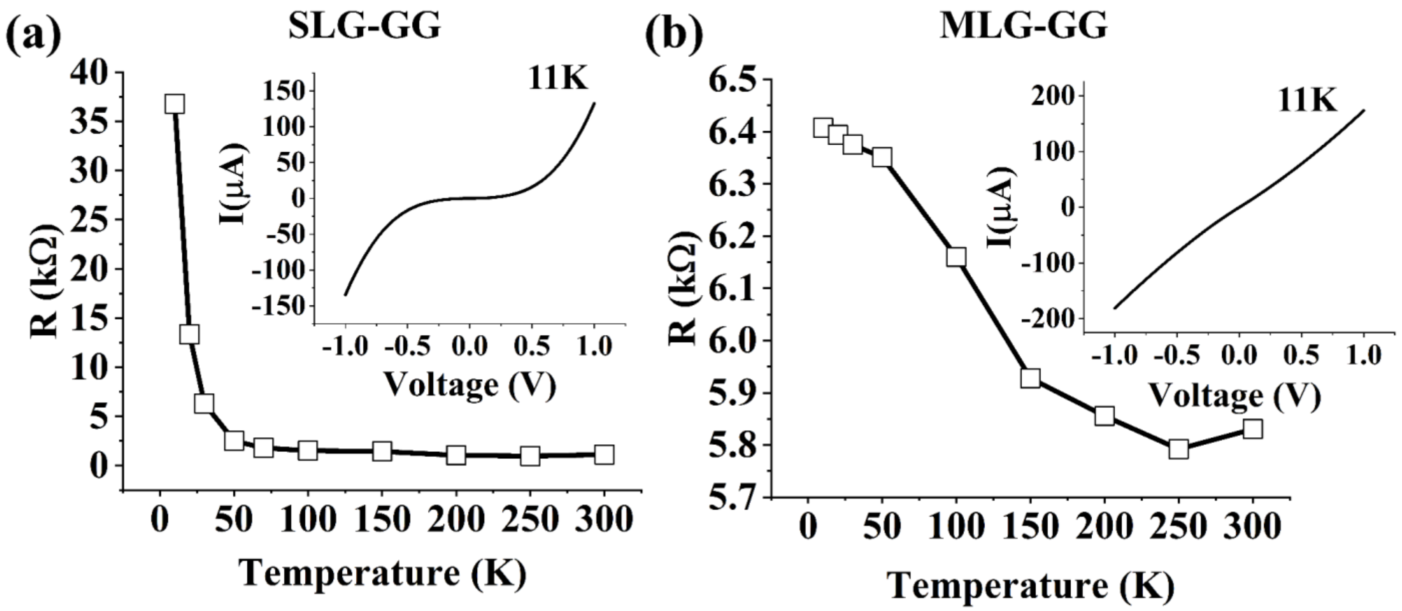

Figure S3. Temperature-dependent resistance (measured at 0.5V) and typical *I-V* characteristics of Fmoc-GG functionalized SLG and MLG samples.

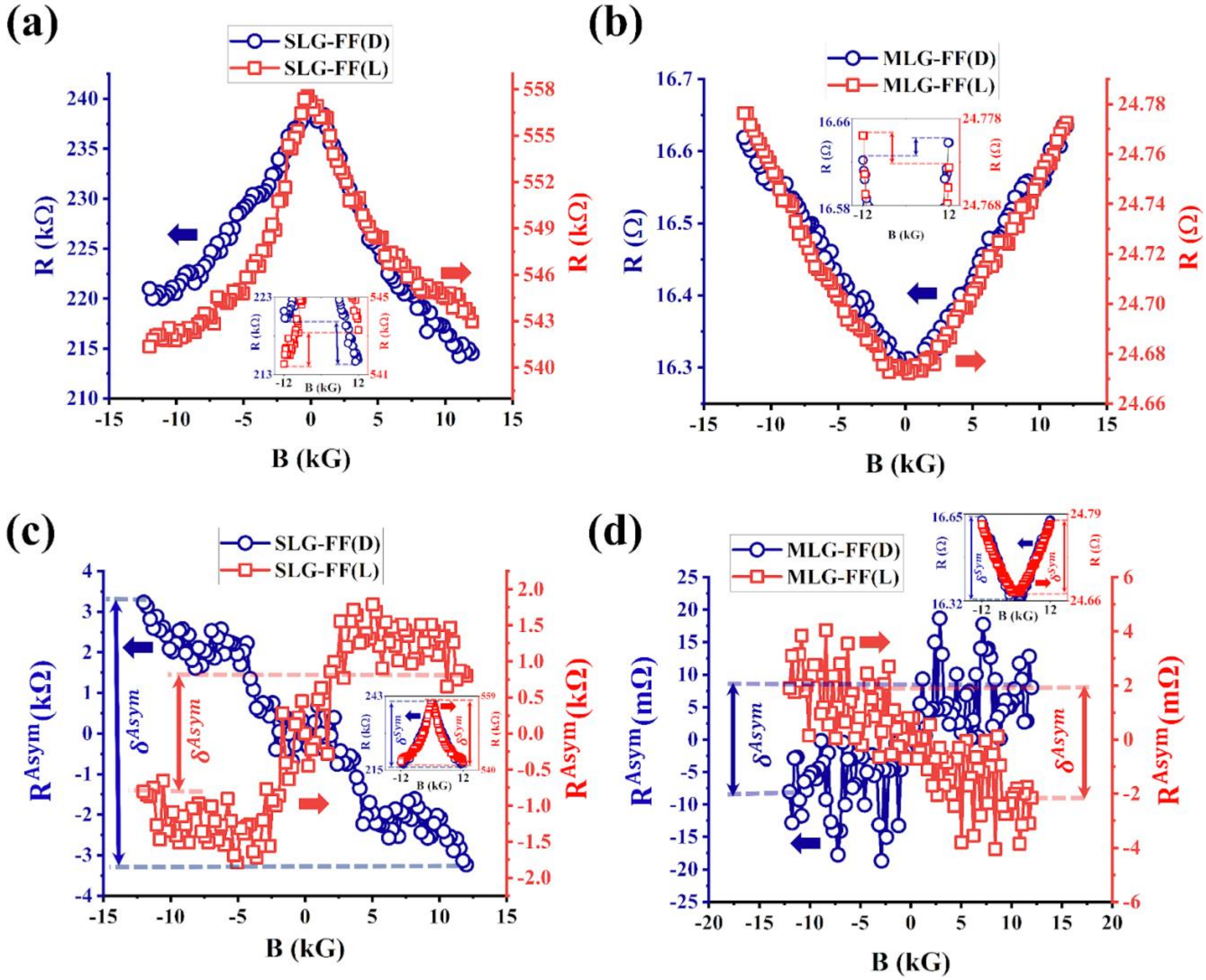

Figure S4. MR data from a different set of samples. (a), (b) *Main images* – MR responses of Fmoc-FF L/D functionalized SLG and MLG samples respectively. The *insets* show the *asymmetry* of the MR responses. Resistance values are measured at 0.5V. (c), (d) Odd (*main image*) and even (*insets*) components of the MR response. The computed CISS signal ($\delta^{Asym}/\delta^{Sym}$) values are as follows: SLG FF D: - 24.9%; SLG FF L: 10%; MLG FF D: 5.1%; MLG FF L: - 3.7%.
